# Introducing a Smart City Component in a Robotic Competition: A Field Report

**DOI:** 10.3389/frobt.2022.728628

**Published:** 2022-02-16

**Authors:** Gianluca Bardaro, Enrico Daga, Jason Carvalho, Agnese Chiatti, Enrico Motta

**Affiliations:** Knowledge Media Institute, The Open University, Milton Keynes, United Kingdom

**Keywords:** smart cities, robotics, robot competitions, robot-city interaction, data hub

## Abstract

In recent years, two fields have become more prominent in our everyday life: smart cities and service robots. In a smart city, information is collected from distributed sensors around the city into centralised data hubs and used to improve the efficiency of the city systems and provide better services to citizens. Exploiting major advances in Computer Vision and Machine Learning, service robots have evolved from performing simple tasks to playing the role of hotel concierges, museum guides, waiters in cafes and restaurants, home assistants, automated delivery drones, and more. As digital agents, robots can be prime members of the smart city vision. On the one hand, smart city data can be accessed by robots to gain information that is relevant to the task in hand. On the other hand, robots can act as mobile sensors and actuators on behalf of the smart city, thus contributing to the data acquisition process. However, the connection between service robots and smart cities is surprisingly under-explored. In an effort to stimulate advances on the integration between robots and smart cities, we turned to robot competitions and hosted the first Smart Cities Robotics Challenge (SciRoc). The contest included activities specifically designed to require cooperation between robots and the MK Data Hub, a Smart City data infrastructure. In this article, we report on the competition held in Milton Keynes (UK) in September 2019, focusing in particular on the role played by the MK Data Hub in simulating a Smart City Data Infrastructure for service robots. Additionally, we discuss the feedback we received from the various people involved in the SciRoc Challenge, including participants, members of the public and organisers, and summarise the lessons learnt from this experience.

## 1 Introduction

Almost 20 years ago, iRobot Roomba (Forlizzi and DiSalvo, 2006) made its debut in our homes as one of the first domestic robots. Today, it is normal to see houses served by autonomous vacuum cleaners, lawnmowers, or floor cleaning robots. These machines have become part of our everyday life (Wirtz et al., 2018) and recent technological advances, such as better vision components, more accurate localisation, optimised energy consumption, and improved machine learning, have extended the scope of service robots from the confined environment of an house to the more open and complex space of the city. Currently, many different robots roam and serve our cities. Particularly popular is the automation of logistics, both for human (Salonen and Haavisto, 2019) and goods transportation (Schneider, 2019). Self-driving pods for tailored public transportation have been trialled in many locations around the world (Wigley and Rose, 2020), autonomous ground delivery robots are active in multiple cities and university campuses (Hoffmann and Prause, 2018) and companies are pushing to introduce flying robots for express deliveries (Ulmer and Thomas, 2018). Autonomous security robots have also been deployed in cities (Loke, 2018). While they provide the same function as security cameras, they also offer an interface that citizens can use to contact emergency services (Lopez et al., 2017). The ongoing healthcare emergency has also showed that robots can be successfully used to support the activities of public authorities in managing and containing emergencies. Examples include robots used to sanitise large public environments (Guettari et al., 2020), as well as robots reminding people about healthcare regulations (Sathyamoorthy et al., 2020).

Nonetheless, even considering all these successful deployments, robots and autonomous agents are still far from being extensively deployed in urban scenarios. In addition, most of those already active have limited functionalities and do not maximise the positive impact they could have on the city (While et al., 2021). This is because the intrinsic complexity and unpredictability of urban environments makes the task of deploying a robot particularly challenging and significantly limits the applicability of service robotics. At the same time, modern cities are exploiting techniques from a wide range of domains, such as pervasive sensors, ubiquitous computing, data analysis and semantic technologies, to become more connected and better adapt to the needs of their citizens (Okai et al., 2018). The connection between a digitalised city and robots has been discussed in (Tiddi et al., 2020). In particular, the authors point out that a single mobile robot equipped with multiple sensors can provide a greater range of data than static sensors. Additionally, robots can exploit the infrastructure of the smart city to support their activities, exchange information with city systems, and access useful environmental data. For example, a delivery robot could use the data coming from a smart traffic light to optimise its route, or the city could adjust dynamically its public transport system based on the number of people using a driverless transport system.

Even though, in principle, the benefits for both sides are evident, there are very few real-world examples of bidirectional interaction between robots and smart cities. There are several reasons for this (Tiddi et al., 2020). First of all, both fields (i.e., urban robots and smart cities) are relatively young and have separate research communities. On the one hand, service robots are typically designed as self-contained and independent devices, able to plan and act according to their own perception. This approach to robotics has led to applications which are robust, but also compartmentalised, i.e., disconnected from the larger technological ecosystem. Smart cities, on the other hand, are expected to address the challenge of effectively reconciling the large-scale data (e.g., power consumption, traffic distribution, etc.) produced by pervasive sensors, including robots. Moreover, reciprocal exploitation is not enough to achieve a full integration of robots in the smart city: it is also necessary to recognise autonomous agents as proactive and interactive elements of the infrastructure. This requires robots to partially expose their internal state to the city, which can monitor and coordinate multiple robotic activities, and provide a robust system to track and analyse the behaviour of robots throughout the city. Moreover, it is important to recognise the role of robots as part of the community by promoting interactions with the general public, both directly, as physical human-robot interactions, and indirectly, by using the smart city as a mean to deliver robotic functionalities to the citizen.

While we recognise the importance of the connection between robots and smart cities, we also acknowledge the challenges of deploying a robot in a complex and interconnected system such as a city. To ease the transition from self-contained robotic platforms with a focused functionality to a robot that is a digital citizen of the smart city, we engaged with the research community organising robots competitions: the European Robotics League (ERL), and hosted the SciRoc challenge, i.e., a competition that simulates the types of challenges faced by roboticists when deploying robots in a city. The first Smart City Robots Competition was held in Milton Keynes (UK) in September 2019.

Robot competitions have long been at the forefront of robotic innovation and have been used to create standardised scenarios to evaluate and benchmark robot capabilities. This article focuses on evaluating the impact of the Smart City concept in the SciRoc Challenge and obtaining some lessons learnt about the development of city-integrated service robots. We do this from four perspectives. The first perspective concerns the role of the smart city in the analysis and design of robotic activities. Unlike previous competitions, a key component of the SciRoc Challenge is the interaction between robots and smart cities. The second perspective relates to the role of a Data Hub within the competition tasks and, indirectly, its impact on the development teams. In particular, we set up a system to simulate the data exchange between the robots and a larger infrastructure, relying on the MK Data Hub, a Smart City research data platform for the city of Milton Keynes. The third element refers to the role of a Data Hub in relation to the public. Specifically, we promoted public engagement both by defining challenges that required direct interaction with the general public and also by visualising and explaining robot activities on screens facing the audience. Finally, the Data Hub can support the long-term preservation and analysis of robot performances. In this case, we provided long term storage of all the data collected during the challenges to give the teams and the organisers a way to analyse the results. Hence, the resulting data infrastructure also provides a baseline for the organisation of future competitions aimed at evaluating the integration of robotic platforms and smart cities.

The rest of the article is structured as follows. The next Section describes the organisation of the first Smart City and Robotic Challenge. Section 3 introduces the MK Data Hub and the components specifically implemented to support the competition. In Section 4, we describe the tasks of the competition in the light of their interaction with the smart city. To evaluate the quality of the solutions developed for the SciRoc Challenge, in particular with respect to integrating service robots in the smart city, we asked organisers, participants and members of the public to fill a questionnaire. We report on the findings of the survey in Section 5. Section 6 discusses lessons learnt and directions for research, while Section 7 summarises the key contributions of this paper.

## 2 Smart City and Robotic Challenge

The European Robotics League (ERL) (euRobotics, 2021) provides a framework to organise robotic competitions at a European level. It is built on the success and legacy of previous European projects, such as RoCKIn (Lima et al., 2016), euRathlon (Schneider et al., 2015), EuRoC (van der Meer et al., 2020) and ROCKEU2, and it is currently run and supported by a Horizon 2020 project, SciRoc (SciRoc, 2021). ERL organises local and major tournaments based in Europe that are open to international participation\enleadertwodots Local tournaments are smaller events where teams are invited to test their robots in certified testbeds across different European institutions, while major tournaments are full scale competitions where multiple teams compete against each other in a bigger event. The challenges proposed by ERL are divided in three categories: service, industrial and emergency. Each category aims at benchmarking and evaluating specific capabilities connected to a category of robots. ERL Service focuses on HRI functionalities and activities in domestic and human-centric environments, such as personal assistants and robotic waiters. ERL Industrial is centred around manipulation tasks and robots in industrial settings, such as pick-and-place and object delivery. ERL Emergency benchmarks robots working in extreme conditions, such as off-road robotics, aerial robots, or marine robotics.

With SciRoc, ERL has expanded its range of competitions to include also the integration of robots within a Smart City. Hence, the first Smart City and Robotic Challenge was conceived as a vehicle for providing (i) a proof of concept of the activities and functionalities expected by a robot in a smart city, as well as (ii) a showcase event bringing an advanced robotic competition to the general public. Specifically, in terms of our work on bringing a smart city element to robotic competitions, we focused on three main objectives: fostering public engagement, enabling robot introspection and interaction, and supporting long-term logging. The latter two objectives are crucial not only to ensure the interaction between the robots and the smart city, but also to increase the robustness and reproducibility of robotic competitions.

Public engagement was fostered in multiple ways. First, the competition was held in a public space, inside the largest shopping mall in Milton Keynes, which is visited, on average, by half a million people per week. Additionally, the general public was directly involved in the competition tasks, through a volunteering program. Finally, multiple screens were used to visualise the behaviour of the competing robots and the evolution of the competition for the benefit of the spectators. Robot introspection and interaction provide the requirements on top of which the integration of robots and smart cities can be built. In addition, in the context of robot competitions, while a referee can observe the behaviour of robots, a more detailed benchmark can be achieved using advanced robot introspection. Finally, one of the elements that makes a city smart is the ability to adapt and improve services by analysing historical data. Therefore, robots need to track and record their activities for long-term logging. Again, this is useful also in the context of competitions, where logged data can be used for replay, analysis and debugging.

These three objectives were achieved thanks to the use of the MK Data Hub, a centralised data infrastructure that we used to simulate the interaction between the robot and the smart city, to collect data generated by the robots and relay it to the public, and to store long-term logs of the activities performed during the competition.

## 3 MK Data Hub

Online platforms for data sharing and reuse, also known as data hubs, are a crucial component of emerging smart cities (Nam and Pardo, 2011; Townsend, 2013). The purpose of these data hubs is to provide a unique access point to the city data, which may include both static datasets (e.g., demographic data) as well as live sensor data (e.g., real-time traffic data, environmental data, and others).

While commercial solutions exist to support the creation of data hubs, we decided to rely on the MK Data Hub (d’Aquin et al., 2015) for this application. The MK Data Hub was one of the major results of the MK:Smart project^1^, a flagship UK initiative that significantly advanced the smart city agenda in Milton Keynes. During MK:Smart, developing an ad hoc infrastructure was motivated by the need to maintain local control over the data to comply with organisational data policies and reduce the costs in the long term. In SciRoc, we decided to continue to use the MK Data Hub since it was already well integrated into the Milton Keynes smart city infrastructure and gave us the necessary level of control and customisation necessary to achieve our objectives.The MK Data Hub provides a state of the art computational infrastructure, which supports the acquisition and management of city data. It comprises solutions for data cataloguing, processing, and delivery, and capitalises on research carried out at the Knowledge Media Institute of The Open University on topics such as policy reasoning and data integration (Daga et al., 2015). Specifically, the MK Data Hub provides both a catalogue of hundreds of data sources, as well as a development environment to facilitate the creation of data-intensive applications (Daga et al., 2016). In particular, three components of the MK Data Hub played a key role in the SciRoc Challenge:

• the Data Catalogue, which supports the creation and management of data sources;
• the Policy Management layer, which supports fine-grained visibility and sharing policies, including a token-based keys management system and access control;
• the API, which supports data management and application development.

These capabilities relate to several aspects of the SciRoc competition. First, the Smart City environment provides the context in which robots operate, allowing them to access and provide data to other agents in a timely manner. The MK Data Hub was used to simulate the smart city environment during the competition: e.g., by providing a coffee shop menu or by keeping track of the status of an inventory.

Second, the MK Data Hub made it possible to monitor and report on the internal status of the robots. Indeed, developers were asked to send human readable explanations of the robot actions illustrating their intentions or decisions. For example, a waiter robot would report on its intention of delivering an order, the number of people sitting in a table, or the presence of a waiting customer. Crucially, these messages allowed the referee to assess the validity of each deliberation.

Third, the continuous data exchange between the robots and the MK Data Hub introduced a novel aspect of public engagement in robotic competitions. Usually, ERL challenges do not involve the general public and are limited to an audience of robotic experts. Therefore, the need to clearly communicate the status and the behaviour of the competing robots was never present. Differently from prior ERL editions, SciRoc was designed to directly involve the public, both spectators and participants. Therefore, it was crucial to guarantee that even non-experts could grasp what was happening during the competition at a glance. We achieved this engagement by exploiting the information collected by the MK Data Hub to show various reports on the screens distributed around the main competition area. The displayed reports included, for instance: 2D maps of the arena, live tracking of the robot position and behaviour, and the outcomes of previous trials. These reports added an essential element of storytelling and understanding to each challenge.

A composite API enabled robots to exchange data with the MK Data Hub. It needs to be noted that the SciRoc rulebooks were developed separately, focusing only on benchmarking requirements and without considering existing capabilities of the MK Data Hub. These requirements were analysed from the perspective of model-driven design in software engineering and a number of data structures were identified and specified as JSON schemas. The resulting API is documented by using the OpenAPI 3.0 specification and the Swagger framework. The methods of the API supported ingesting and querying the data according to previously defined data schemas, supporting standard CRUD operations, and a general purpose JSON-based query language, mirroring the features of the underlying database (MongoDB). The MK Data Hub functionalities were made available 2 months before the competition to the participating teams to enable them to start prototyping and optimising their software ahead of the event. In addition, thanks to the policy management layer of the MK Data Hub, each team could access a shared data environment without the risk of modifying or corrupting information outside the provided boundaries (Daga et al., 2016).

For each episode, the MK Data Hub provided reference data and monitoring capabilities. All robots were connected to the MK Data Hub and could send their location and status messages. These could be displayed in the showcase screens for monitoring but also to entertain visitors. Having the location on the MK Data Hub allowed us to display the arena on screens and monitor the path performed by the robots. Crucially, these data allowed us to “replay” trials after the competition, by mixing data sent by the robots with a map of the arena and synchronising it with a video capture.

The functionalities of the MK Data Hub were provided by four applications.

**FIGURE 1:**
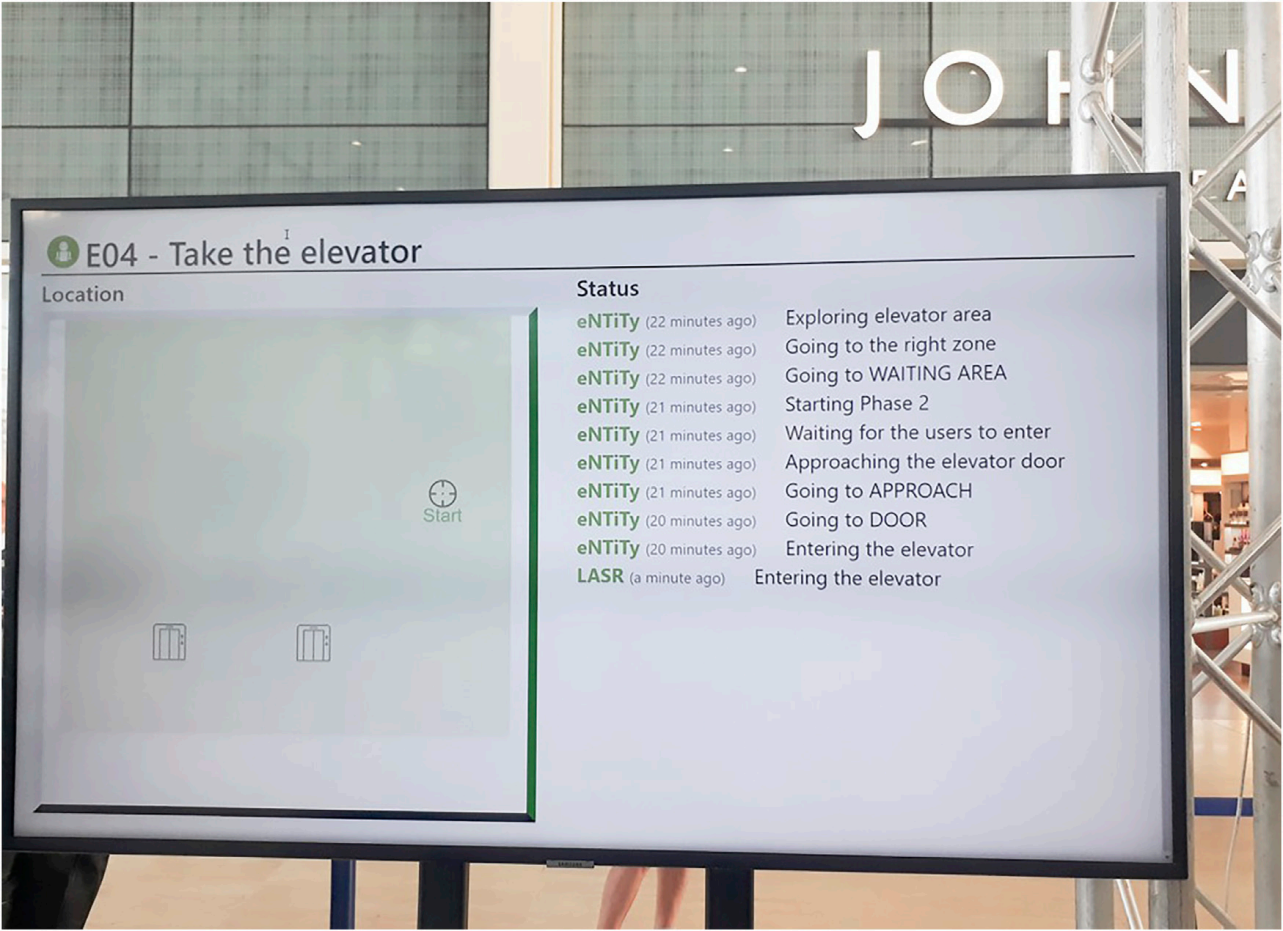
This picture, taken during the competition shows one of the screens in action. In particular, it shows the current status of a robot during a trial of the “Take the elevator” challenge. On the left hand side, a live map of the episode is shown, while the right hand side displays status messages sent by the robots.

**FIGURE 2:**
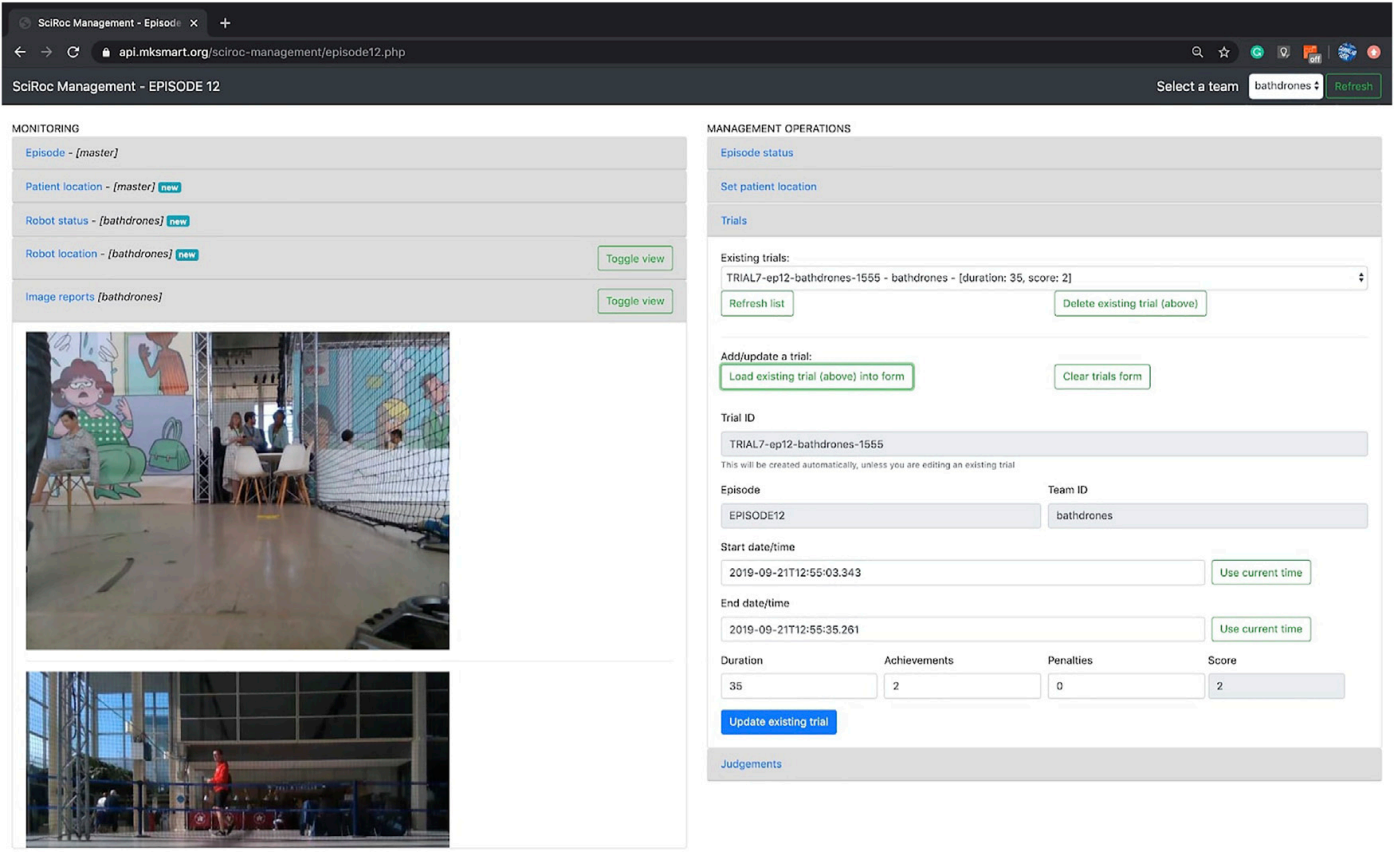
A screenshot of the Trial Management Interface for the “Fast delivery of emergency pills” challenge. The left hand side shows information related to the trial, which was provided by the Data Hub (e.g., Patient Location) and the robot (e.g., live pictures). The right hand side shows instead the interface used by referees to record the results of the trial.

**FIGURE 3:**
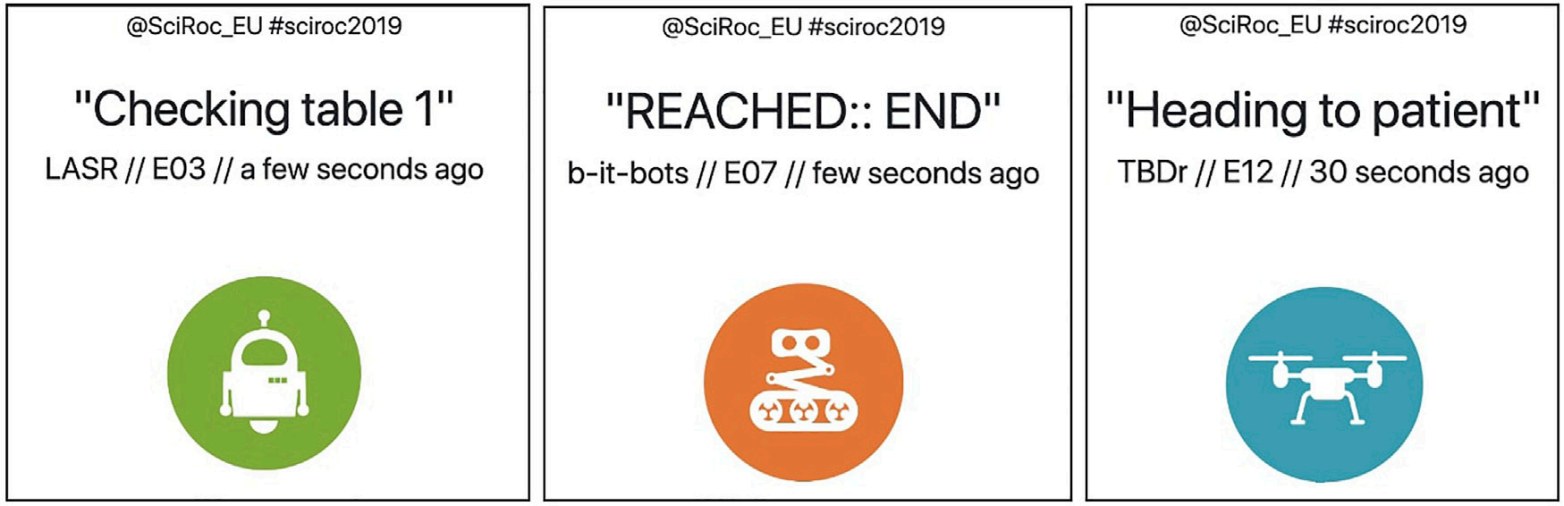
An excerpt of the SciRoc Showcase. This is an aggregate view of the active trials among all challenges. Each box shows the most recent status message sent by a specific team in a specific challenge.

**FIGURE 4:**
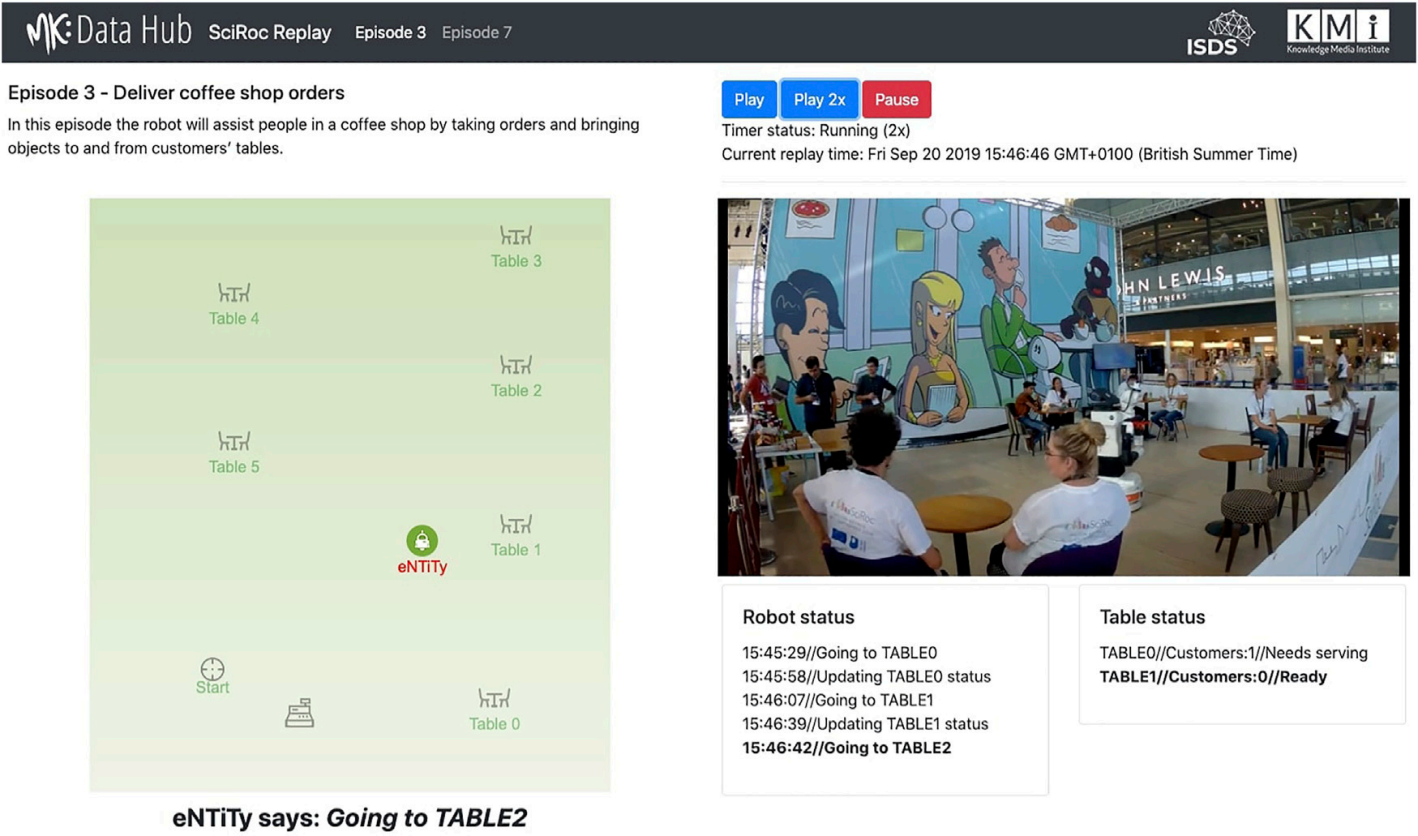
Interface of the SciRoc Replay Demo for the “Deliver coffee shop orders” challenge. The left hand side shows the map of the arena with the current position of the robot and its most recent status message. On the right hand side, we can see a synchronised video of the trial showing the robot in action.

• SciRoc Monitor interface. Displayed on the screens during the competition, the monitor interface allowed the audience to follow the trials from a data-driven point of view, displaying both the location of the robots in the arena and real-time status messages. See Figure 1 for an example from the competition.
• SciRoc Trial Management interface. This system was used by the assistant referees to assist the referees of each challenge in their evaluation of the behaviour and performance of the robot. The interface had two main areas: monitoring and management operations. In the monitoring section, referees and assistant referees had access to tailored information about the current active challenge (e.g, real time maps, measurements collected by the robot). The assistant referees used the management operation area to setup basic information about trials, such as start time, end time, and related team. Additionally, they were provided with tools to reset the status of the Data Hub before each trial. Finally, through the Trial Management Interface (TMI), the Data Hub was used to record the results of each trial, such as the final score and the total execution time. Figure 2 shows an example of the TMI.
• SciRoc Showcase. The SciRoc Showcase was a general purpose, customisable collection of static and real-time views over the competition (Figure 3 shows one example), displayed on screens in various locations. The Showcase included an overview of the competition, teams and sponsors, a description of each episode, and real-time information such as views from the monitor interface, messages from robots, and an updated summary of trials and scores for each team and episode. Essentially, the SciRoc showcase constituted an anthology of facts and events collected in real-time for the sake of supporting participants and informing visitors.
• SciRoc Replay Demo^2^. The data collected during the competition allowed us to reconstruct two trials for which we also had a complete video recording. This demo demonstrates the potential of using a Data Hub in robot competitions not only to simulate a smart city component but also to record a thorough, end-to-end account of the trials. Figure 4 shows the interface of the SciRoc Replay.

Deploying a data ecosystem in the competition opens up opportunities but also brings its own additional requirements. Primarily, it is of crucial importance that the capabilities of the Wi-Fi local network(s) permit a high volume of data traffic.

## 4 The Challenges

**FIGURE 5:**
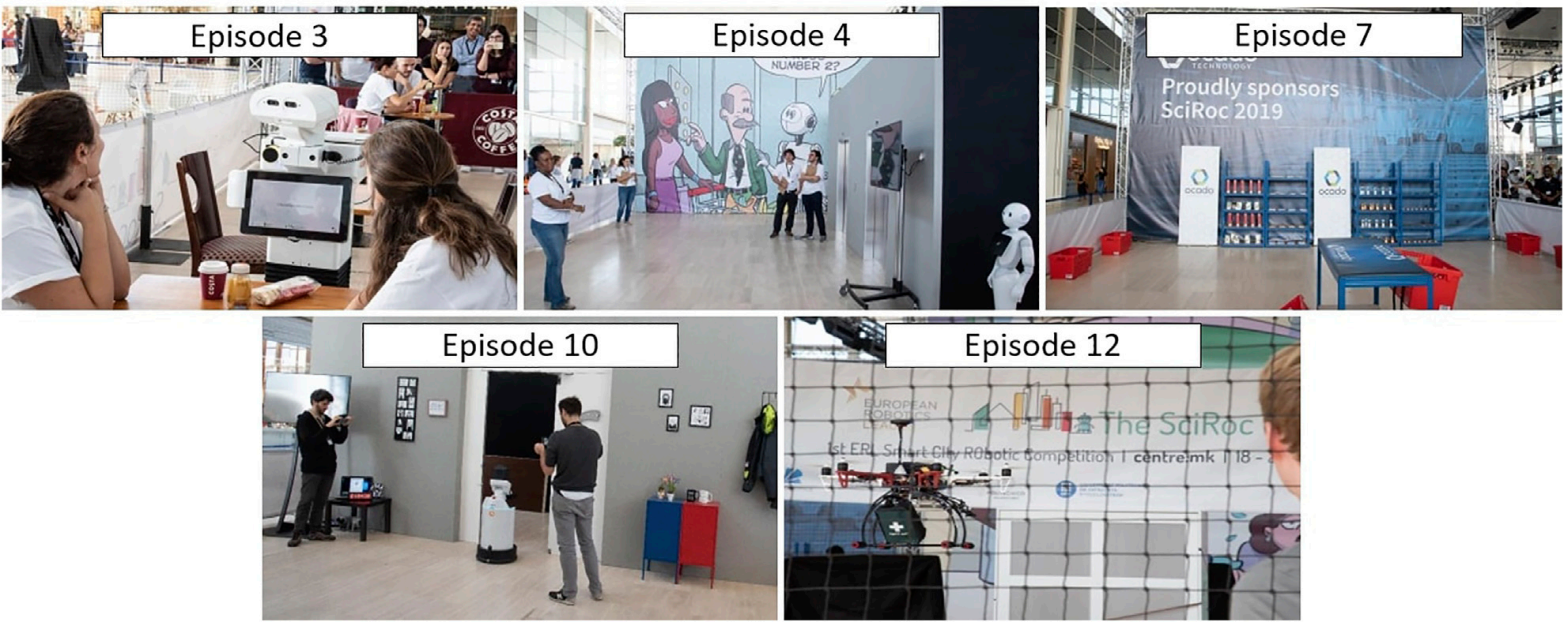
Pictures taken during the competition showing different robots engaging in the various challenges.

In SciRoc, teams compete in different challenges created to benchmark a specific set of functionalities. To make them more understandable by the general public, each task was framed in a story representing an interaction between the robot and the user or the smart city. For this reason, each of these tasks is called an episode. The original set of episodes contained twelve different tasks, focusing on specific macro activities (e.g., manipulation, flying robots, social navigation, etc.). As part of the definition of the rulebooks of the episodes, each technical committee highlighted potential ethical issues associated with the presence of human participants and how to avoid them. The rulebooks were reviewed and approved by an ethics committee to ensure no issue was present or left unmitigated. Out of these twelve tasks, five were selected after feedback from the early-registered teams (see Figure 5).

**FIGURE 6:**
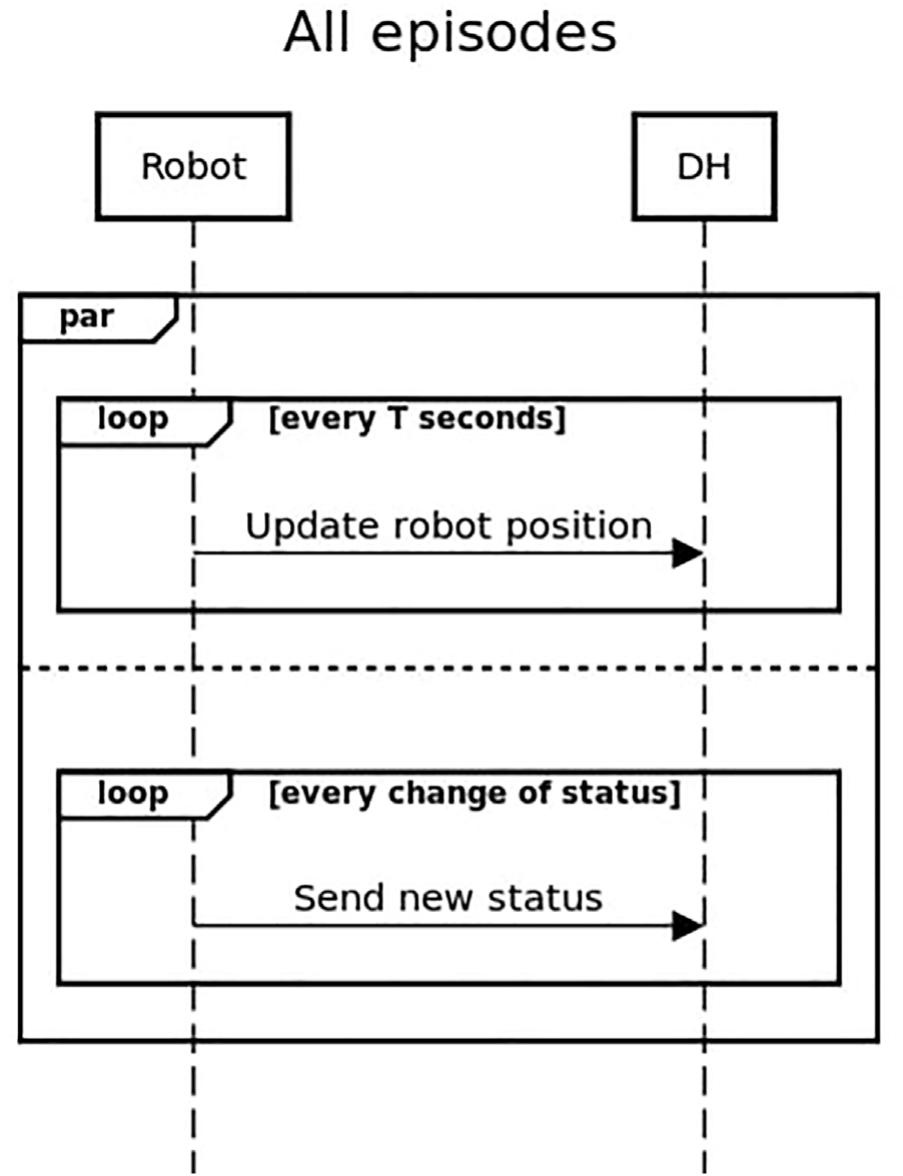
Sequence diagram showing the general interaction between the robot and the Data Hub. This is valid for all episodes.

Each episode has two aspects, the physical challenge for the robot and the interaction with the smart city. The first aspect is the direct interaction of the robot with the physical world or the human participants. While, the second aspect is represented by a data exchange between the robot and the MK Data Hub. As described in the sequence diagram in Figure 6, during the execution of the challenges the robot will have to constantly update the Data Hub with their current location, and pair each of their actions (e.g., met a customer, opened the door, etc.) with a status message. This information was used by the SciRoc monitor interface to keep the public updated on the activities of the robot, and by the referees through the SciRoc trial management interface to assess the correctness of the execution and score the episode.

In the following, we give a brief description of each episode, focusing mostly on the interaction with the smart city component. A complete and detailed description of the challenges is available as a public deliverable^3^ of the project. The deliverable contains the full rulebooks used to define each episode. The rulebooks are written by a technical committee of experts in the theme of each challenge (e.g., human-robot interaction, manipulation, aerial robotics, etc.). The rulebooks provide a fully defined description of the episodes including precise details of the arena, any constraint on the robot platform to be used, and the list of achievements used to evaluate the competitors.

### 4.1 Episode 3—Deliver Coffee Shop Orders

In this episode the robot assists the staff of a coffee shop to take care of their customers. The robot is asked to perform multiple subtasks in a specific order during the episode. Specifically, it needs to keep the status of the tables inside the shop updated (e.g., “empty”, “with people”, “to be cleaned”, etc.), take orders from the waiting customers, deliver objects to the tables, detect a waiting customer, and guide him/her to an empty table.

The episode arena is a predefined rectangular area fitted to resemble a coffee shop. A total of six tables and a variable number of chairs are present in the arena during each trial. Additionally, at the entrance of the arena, two tables are arranged to replicate a counter: an area where the new customers wait and where the robot receives the object to be delivered to the tables. The location of the main furniture elements (i.e., the counter and the tables) was fixed during the entire competition, and the teams could pre-record a map of the environment for navigation. Since the shape, the size and the position of the furniture was constant during the competition, these elements were also stored in the Data Hub and presented on screen to the general public. Chairs are considered as mobile obstacles; hence, they were not included in the mapping phase. To create a more realistic setup, the arena was fitted using the same tables, chairs and banners used in a real Costa coffee shop. Moreover, the products ordered by the customers and delivered by the robot were selected from a set of Costa products, such as coffee cups of different sizes, sandwiches, and bottles of juice.

**FIGURE 7:**
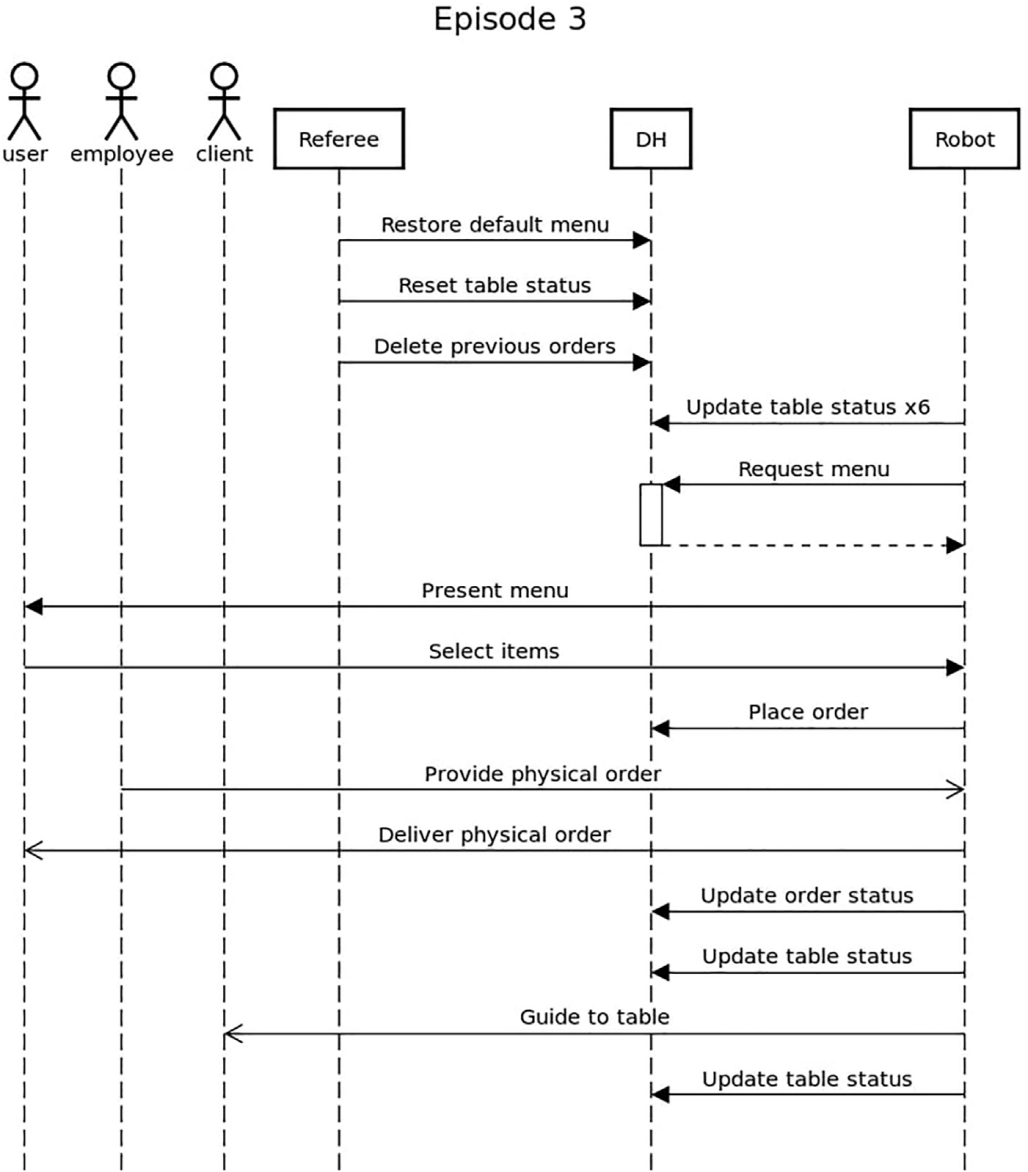
Sequence diagram showing the interaction between the robot and the Data Hub for Episode 3

The execution of this episode is divided in three phases and includes multiple interactions with the Data Hub. A schema of the interactions between the users, the robot and the Data Hub is shown in the sequence diagram in Figure 7. In the initial phase the robot is tasked with the identification of the initial condition of the coffee shop. To better simulate a real scenario, an entry for each table is already present in the Data Hub at the beginning of the trial. However, this default entry contains invalid data, to avoid randomly selecting a correct configuration and giving unfair advantage to a team. After assessing the status of the coffee shop, the robot needs to identify an empty table (i.e., no items on it) with at least one customer (i.e., the customer is waiting to order). This can be done both using an internal representation stored by the robot, or by querying the Data Hub. The next phase of the episode is the interaction with the customer at the table. The robot requests an up-to-date menu from the Data Hub and present it to the customer using its preferred interface (e.g., a screen, dialog, etc.). Then, it receives from the customer a list of products, again using any interface. The order has to be correctly uploaded on the Data Hub and will be used by the referee to provide the products to be delivered. The robot collects the items from the counter and delivers them to the correct table. At the end of the delivery, the order is marked as completed on the Data Hub. During the entire process, the robot is expected to keep the status of the table constantly updated. The third phase of the episode consists in guiding a new customer to an empty table. The robot has to greet the customer, identify a suitable table (using the Data Hub or an internal representation), and then guide him/her to the table. The performance of the robot is ranked not only based on its capability of solving the physical task, but also based on the consistency between the information logged on the Data Hub and the robot’s actions.

### 4.2 Episode 4—Take the Elevator

The episode revolves around the activity of taking an elevator. Often service robots do not have the correct hardware configuration to be able to interact with a normal elevator, therefore this simple task requires many capabilities to be completed. For instance, this task requires the robot to interact with people and to ask them to select the correct floor. Moreover, this task implies that the robot understands what floor the elevator is currently at.

The arena of the episode is divided in two areas: a wooden structure representing the mock-up elevator and an L-shaped space for the robot to navigate. The mock-up elevator has a movable door manually controlled by a human operator inside the scaffolding. Using a camera, the operator can monitor the execution of the episode and open or close the door when the human volunteer interacts with the mock-up elevator. There is no screen or highlighted button inside the elevator to show the current or destination floor. This design put the focus on human-robot interaction through dialogue instead of using visual cues to collect information.

**FIGURE 8:**
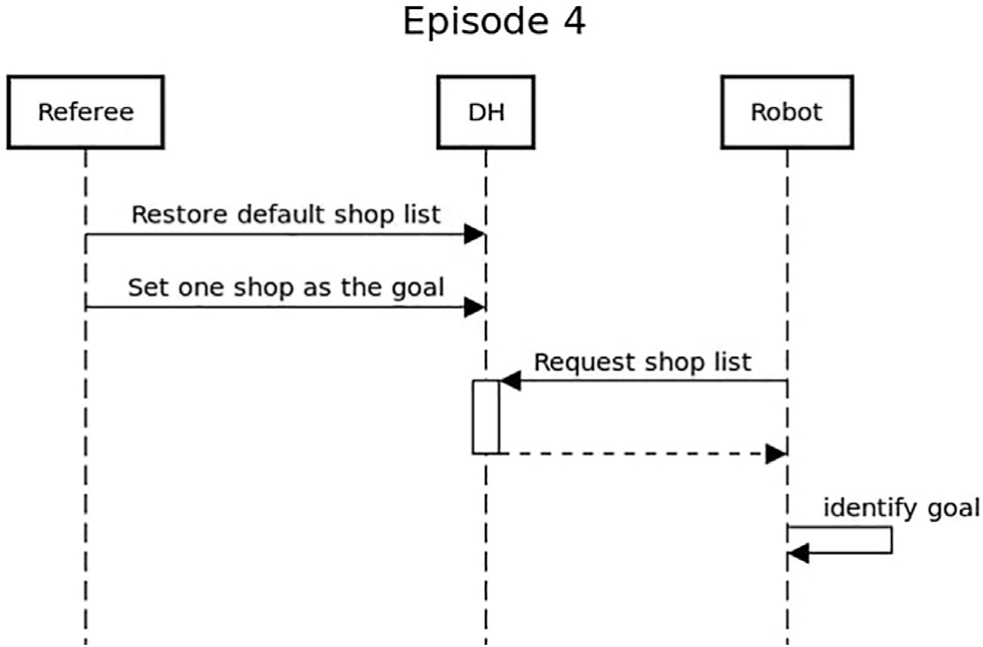
Sequence diagram showing the interaction between the robot and the Data Hub for Episode 4

The episode is divided in three phases. The episode starts with an interaction with the Data Hub, the robot requests the list of possible destinations (i.e., shops with a corresponding floor) and identifies one marked as the goal. The flow of this interaction is represented by the sequence diagram in Figure 8. Given the strong focus on human-robot interaction, no other episode-specific interaction with the Data Hub is expected by the robot. However, as in any other episode, the robot must periodically provide its position and status notification. The initial phase consists of the robot moving from the starting area to the waiting zone in front of the elevator, while avoiding non-interacting humans. This phase concludes when the robot reaches the elevator; this must be notified with a status update on the Data Hub. The next phase starts with the operator manually opening the doors of the elevator. The robot can enter the enclosed area after all the human volunteers and must respect the users’ personal space while in the elevator. When in the elevator the robot has to interact with one of the volunteers to request the selection of the target floor. The trial continues with the door opening multiple times and volunteers leaving the area at any point. The robot has to leave the elevator only at the target floor and always after the human participants. The phase ends with the robot leaving the elevator, announcing the completed sub-task and sending a status message to the Data Hub. In the last phase the robot has to reach the ending area at the opposite end of the arena.

### 4.3 Episode 7—Shopping Pick and Pack

This episode recreates an automated shop where a customer requests an order from a minimal storefront (e.g., a tablet) and a mobile manipulator collects and packages the products for them. The robot receives the list of items, navigates to the predefined locations where they are stored, collects them, and then delivers them to the packing area.

The arena of this episode is a pre-defined area with two main points of interest: the items drop location and the shelving area. The former is a mock-up counter, with a public facing side and two bins used by the robot to deliver the selected products. The latter consists of four shelving units with three shelves each, one at ground level, one at middle height and one at the top. Given the significance of these location, their position is stored in the Data Hub and shown on the visualised map.

The arena is built to accommodate for two different robotic platforms: a smaller mobile manipulator (e.g., Kuka youBot), and a tall semi-humanoid platform (e.g., PAL Robotics Tiago). This is reflected by the different shelving heights and by the two bins at the counter, one at ground level and the other on top of the table.

**FIGURE 9:**
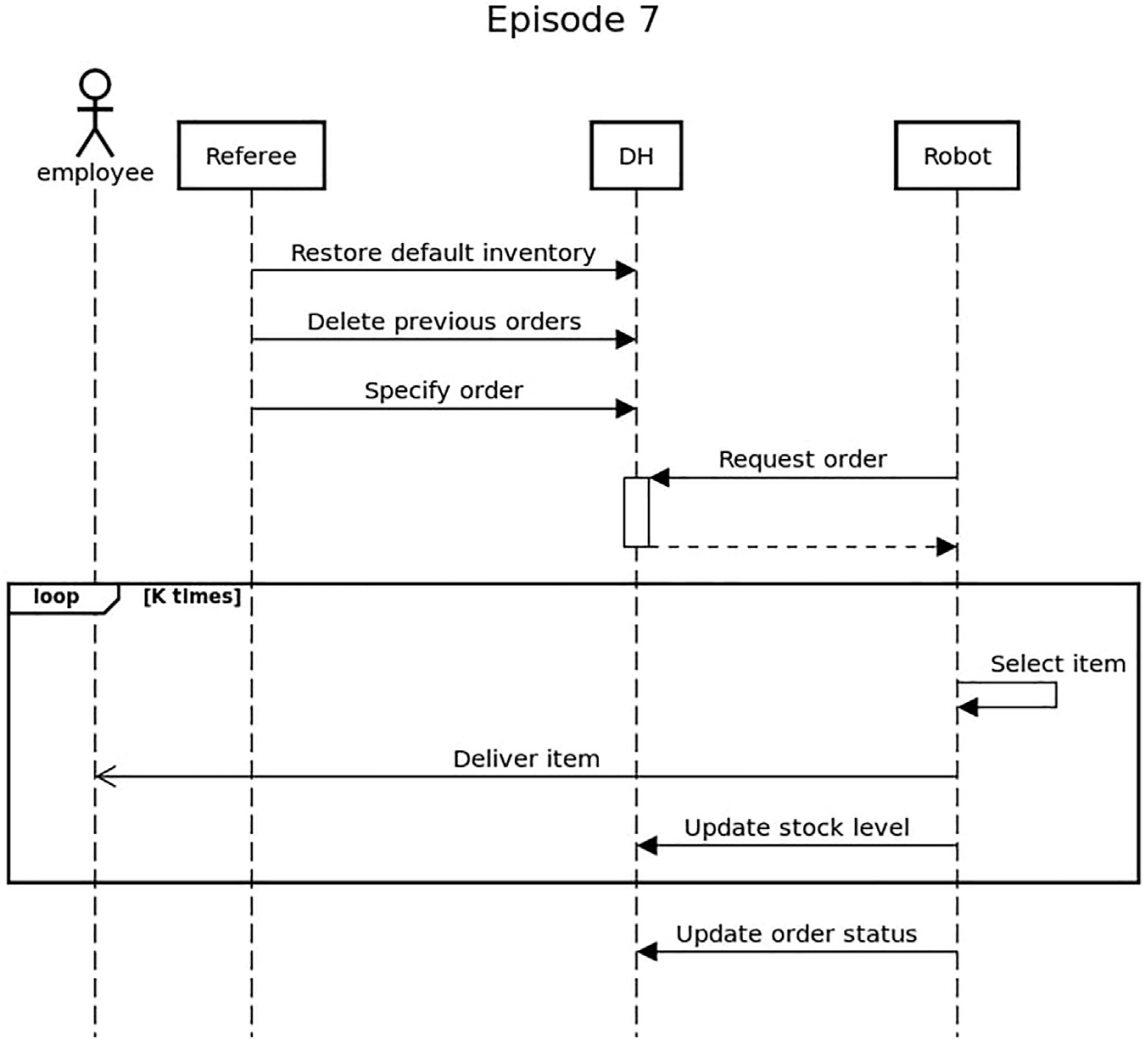
Sequence diagram showing the interaction between the robot and the Data Hub for Episode 7

The episode consists in a setup phase and in multiple delivery actions. The flow of the episode and an overview of the interactions with the Data Hub is represented in Figure 9. During the setup phase the products are placed on the shelves and their quantity and location (i.e., which shelving unit and which shelf) is loaded in the Data Hub. At the beginning of the episode the inventory status stored in the Data Hub is always correct. The robot is placed in the starting area of the arena, which is also the location it has to reach to complete the task. The episode starts when a new order, composed by a list of items each with a specific quantity, is uploaded on the Data Hub. The robot receives the order and start delivering the items one by one. After each interaction with the physical inventory, the robot must update the status of the Data Hub. This means that if the robot collects an item from a shelf and then drop it during the delivery, it has to recognise the missed delivery and update the stock level. After successfully delivering all the products, the robot has to mark the order as completed on the Data Hub and can reach the ending area to conclude the episode.

Consistency between the action of the robot and the status of the Data Hub is extremely important in this episode. Therefore, teams are scored based on the ability of the robot to deliver the objects, but also on the consistency between the action of the robots and the status of the Data Hub.

### 4.4 Episode 10—Through the Door

This episode evaluates the capability of a robot to interact with one of the most ubiquitous devices found in human environments: the hinged door. The challenge requires the robot to approach a closed door, open it, go through and then close it behind itself. While the task appears to be quite simple, doors are extremely challenging to use for any non-standard actor, such as robots, children or disabled people, because they have a very long history of development over which the only design constraints were cost and fitness for human usage.

The arena of this episode is partitioned in two by a separation wall. This wall act as a scaffolding to hold the instrumented door used in the episode. The door is motorised, not to open automatically, but to oppose a force to the user and simulate the behaviour of multiple real doors (e.g., heavy door, object behind, object blocking the door, etc.). Moreover, it is equipped with multiple sensors to detect the interaction with the robot and respond accordingly.

Given the nature of the episode, the high-level description of the activities of the robot is quite straightforward. The robot starts in a designated area on one side of the arena, the objective is to reach the ending area on the opposite side going through the door. In order, the subtasks of the robot are: identify and reach the door, detect and interact with the handle, open the door, go through, close the door, and lock it. The episode ends when the robot reaches the ending zone or when the time runs out. The episode is unique regarding the interaction with the Data Hub, because it does not have any specific message exchanged, since the challenge is purely mechanical. Nevertheless, the robot must follow the general interaction and continuously provide its position and regularly update the Data Hub with status messages. The use of status messages is particularly important in this type of episode since they show the intent of the robot and can be used to differentiate between hard-coded solutions and actual interactions.

### 4.5 Episode 12—Fast Delivery of Emergency Pills

This episode is designed to test the capabilities of aerial robots, or drones. It simulates an emergency situation where a flying robot has to deliver a package containing medical supplies to a specified location. The robot also must provide a visual feedback of the general area and of the emergency situation. Since the episode takes place indoor, the robot cannot rely on Global Navigation Satellite System (GNSS) support for localisation.

For safety reasons and to give to the drones the necessary space to manoeuvre, this arena is significantly larger than the others. Additionally, to protect the public and avoid accidents, the entire space is enclosed with a protective netting. The space of the arena is occupied by furniture (e.g., tables, chairs, cupboards, etc) as ground obstacles, and banners spanning the width of the arena as aerial obstacles.

**FIGURE 10:**
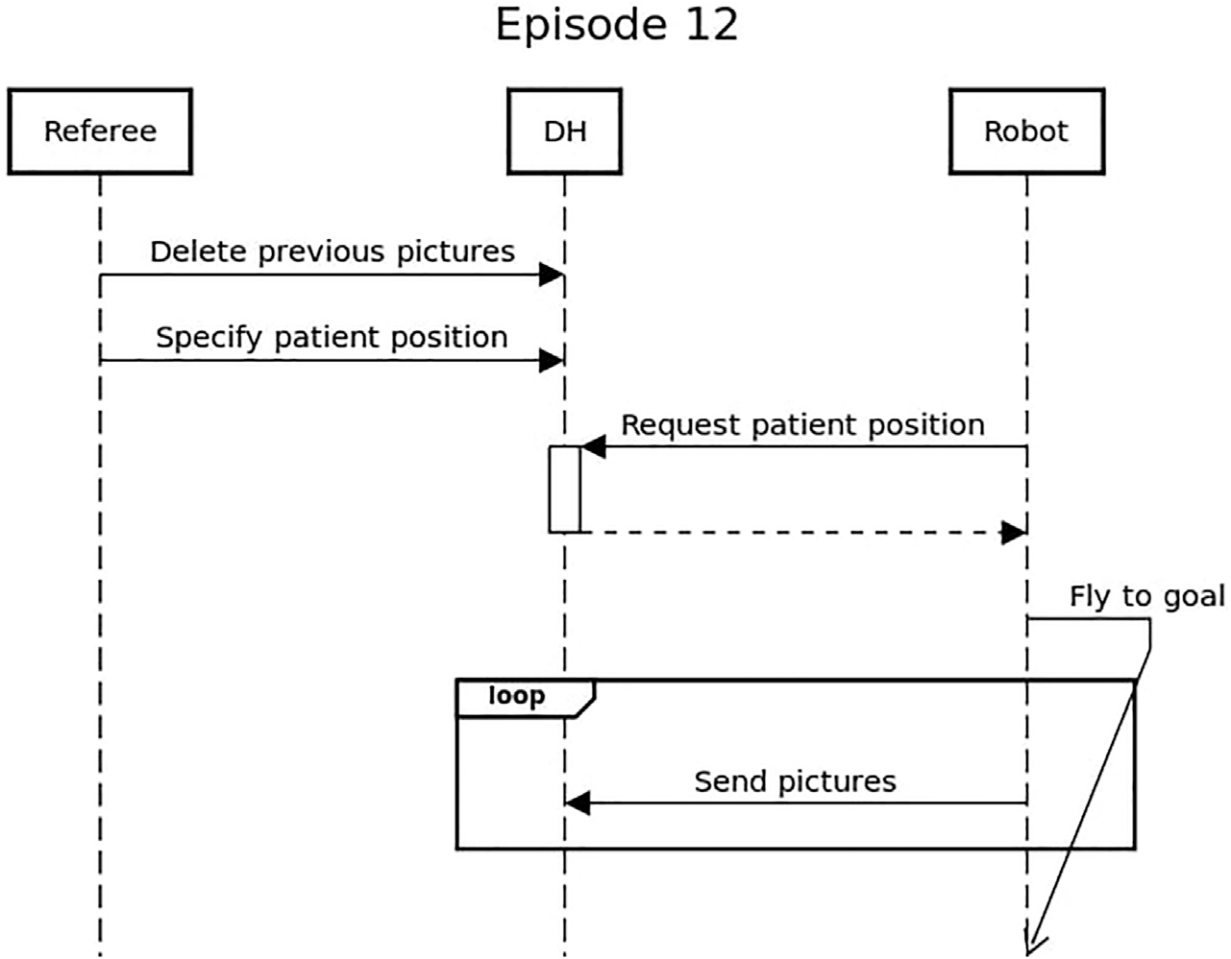
Sequence diagram showing the interaction between the robot and the Data Hub for Episode 12.

The execution of the episode is divided in a setup phase and three execution phases. During the setup the drone is placed in the starting area and the payload is connected to it. The setup ends when everyone leaves the arena with the exception of the safety pilot and one referee. The trial of the episode starts with the robot retrieving from the Data Hub an estimated location of the person in need, represented by a mannequin. The three execution phases proceed as follows: phase one, take-off and flying to the specified location; phase two, landing close to the mannequin and disconnect the payload; phase three, flying back to the starting location. As shown in Figure 10, during all the execution phases, the robot is required to periodically transmit to the Data Hub pictures taken from the environment paired with the current location of the robot. The drone is scored based on the ability of completing each round trip with no collisions, the distance between the mannequin and the dropped payload, and the ability to consistently transmit information to the Data Hub.

## 5 Feedback From the Competition

In the 2019 edition of SciRoc, ten teams from various European universities and companies took part in the competition, for a total of sixty-two participants. Additionally, we recruited forty volunteers with no previous experience in robotics. The volunteers acted as human participants in those episodes where human-robot interaction was present. Finally, various experts were involved in the organisation of the competition, definition of the rulebook and scoring of the trials.

To assess how the various actors involved in the competition felt about the organisation and the technologies used, we designed a survey to collect various types of feedback.

### 5.1 Structure of the Survey

The survey ^4^ was targeted to the three main roles involved in the competition: referees, competitors, and volunteers. The “referees” category includes anyone that was directly involved in the organisation and running of the competition, such as referees evaluating the performance of the robots, assistant referees managing the interaction with the Data Hub, and organisers working on the design of the challenges and the rulebooks. The competitors are all the members of the teams that took part in the competition. For the questionnaire, we did not consider the participants as part of a team, because we wanted to gauge each competitor’s personal experience. Lastly, volunteers are all the members of the general public that had an active role in the competition by taking part in one or more episodes. All the participants gave their written consent to collect data via a survey as part of their involvement in the competition.

Given the different role, expertise, background, and expectations of each category of participants, we divided the questionnaire into general topics, and then, for each topic, we designed one or more questions that were compatible with the role of each actor in the competition. The first part of the questionnaire was the same for all participants, it included questions on general public engagement, the importance of explanation, monitoring and reproducibility, and how much these were supported in SciRoc. The questionnaire also included a brief explanation of these three concept to ensure that all participants, independently of their background, could answer the questions. Explanation represents being able to understand the intent of the robot and the reasoning behind its behaviour (e.g, travelling from A to B to reach the goal in B). Monitoring is showing a direct report of the actions of the robot (e.g., position of the robot in time). Reproducibility means having enough information about the robot and the environment to replicate the action of the robot. This part also included a question on the screens used to show information about the various competitions, in particular eliciting how useful they were seen by the different categories of people involved in the SciRoc Challenge. A summary of the results for this part of the questionnaire can be found in Table 1.

**TABLE 1:** Summary of the results of the part one of the questionnaire. The table includes the mean (M) and the standard deviation (SD) of each question. The values are aggregated globally and by actor.

	Global		Referees		Competitors		Volunteers	
	M	SD	M	SD	M	SD	M	SD
Engagement of the general public	4.00	0.78	4.00	0.76	4.08	0.83	3.89	0.74
Importance of Explanation	4.36	0.67	4.57	0.62	4.38	0.49	4.00	0.82
Importance of Monitoring	4.19	0.74	4.29	0.70	4.38	0.62	3.78	0.79
Importance of Reproducibility	4.17	0.80	4.21	0.86	4.23	0.70	4.00	0.82
Support to Explanation	3.72	0.77	3.86	0.74	3.77	0.70	3.44	0.83
Support to Monitoring	3.94	0.85	4.29	0.59	4.08	0.83	3.22	0.79
Support to Reproducibility	3.56	0.83	3.21	0.86	4.00	0.55	3.44	0.83
Usefulness of screens	3.83	0.93	3.82	0.83	3.77	0.97	3.44	0.83

The second part of the questionnaire was reserved for referees and competitors because it is centred around the tools provided to them and the design of the competition. In particular, we asked them to evaluate how useful the tools (i.e., APIs for competitors and Trial Management Interface for referees) we provided were and how difficult it was to integrate them into existing workflows. Additionally, we investigated how impactful the Data Hub was in the definition of the rules. Lastly, we asked competitors and referees to identify which elements of the framework we created to support the competition were more useful, and how we could expand them in the future. The results of this part are summarised in Table 2.

**TABLE 2:** Summary of the results of the part two of the questionnaire. The table includes the mean (M) and the standard deviation (SD) of each question. The values are aggregated globally and by actor.

	Global		Referees		Competitors	
	M	SD	M	SD	M	SD
Usefulness Of TMI	3.86	0.64	3.86	0.64	−	−
Usefulness Of APIs	3.69	0.82	−	−	3.69	0.82
Difficulty With Addition Of DH	3.00	0.93	3.00	0.93	−	−
DH Improve The Rules	3.22	0.74	3.14	0.83	3.31	0.61
Difficulty Integration DH	3.31	0.82	−	−	3.31	0.82
DH More Difficult Rules	3.44	0.83	3.71	0.70	3.15	0.86
Usefulness of Maps	4.08	0.86	4.15	0.95	4.00	0.74
Usefulness of Status Messages	4.32	0.68	4.50	0.63	4.09	0.67
Usefulness of TMI	3.80	0.69	4.00	0.65	3.55	0.66
Usefulness of Robot Activity Log	4.13	0.83	4.25	0.83	4.00	0.82

### 5.2 Results Analysis

In total, we received 36 answers to the questionnaire^5^. Out of these 36, 14 were referees, covering all five episodes. Members of the competing teams submitted 13 answers, mostly covering “Episode 3–Deliver coffee shop orders” and “Episode 4–Take the elevator”, since they were the most popular episodes. Lastly, volunteers contributed with nine answers. We will now give an overview of the results we obtained, taking into account the role each actor had in the competition. In the following sections, we report the results in two formats. We use the average paired with standard deviation for the questions that use a Likert scale (from 1 to 5) as the answer. Moreover, we report the percentage of positive answers for binary questions (i.e., yes or no).

#### 5.2.1 Engagement of the General Public

Independently from the role they had in the competition all participants to the questionnaire gave a positive evaluation (4.00± 0.78) of the level of engagement from the general public.

#### 5.2.2 Importance of Explanation, Monitoring, and Reproducibility

Given their different backgrounds, we were expecting different results coming from volunteers (i.e., general public) and people directly involved with the competition (i.e., academics). In fact, on average, volunteers gave a lower importance to all aspects, never above 4.00. Nevertheless, all participants rated explanation (4.36± 0.67) as the most important aspect, but only with a small margin with respect to the others (monitoring 4.19± 0.74 and reproducibility 4.17± 0.80).

#### 5.2.3 Support of Explanation, Monitoring, and Reproducibility

In this case the three different roles gave three different feedbacks. Referees recognised monitoring as the most supported feature (4.29± 0.59), while they were partially unsatisfied by the support given to reproducibility (3.21± 0.86). The teams equally recognised monitoring and reproducibility as the most supported features (4.08± 0.83 and 4.00± 0.55), but also considered the support to explanation more than sufficient (3.77± 0.70). The volunteers gave, on average, lower scores across the board: explanation at 3.44± 0.83, monitoring at 3.22± 0.78, and reproducibility at 3.44± 0.83. This can be traced back to the fact that the general public usually have higher expectations toward technologies with respect to reality. However, the results are all above “Neutral”, highlighting a positive experience of the volunteers during the competition.

#### 5.2.4 Usefulness of Screen

Screens showing multiple information about the robot and the competition provided the main interaction channel between the Data Hub and the public. In general, the participants to the questionnaire gave a positive feedback about the screens, with a global average value of 3.83± 0.93. However, it is necessary to analyse the result by category, since the interaction with the screens was significantly different depending on the role.

The referees gave a positive feedback on the usefulness of the screens, with the highest average value (3.82± 0.83). Referees normally use their own experience in the field to assess the correctness of the behaviour of the robot. Nevertheless, during SciRoc 2019 they could rely on the feedback provided by the screen as a supplementary tool.

The team members also gave a very positive feedback about the screens, however they had the widest range of results (3.77± 0.97). This may be connected to how many tools a specific team already had to monitor the behaviour of the robot. A team with an already available map and monitoring system would have little use of the information provided by the screen, while other teams relied on the screens to visualise the status of the robot and its location in the episode arena.

While maintaining a positive feedback, the volunteers gave, on average, the lowest score to the usefulness of the screens (3.44± 0.83). This may be related to the fact that volunteers were directly involved only in episode three and episode 4. These were also the two episodes with the strongest human-robot interactions. Here the volunteers were more directly involved with the robots, in comparison to observing the episode evolution through the screens.

#### 5.2.5 Usefulness of the Trial Management Interface

The Trial Management Interface (TMI) is a tool specifically designed to support the activities of the referees. In each episode, an assistant referee was in charge of operating the TMI directly, following the instructions of the main referee. Most of the referees appreciated the addition (3.86± 0.64) and considered it useful for their work and a significant upgrade with respect to the paper sheets used in previous competitions.

#### 5.2.6 Usefulness of the APIs

Often in competitions it is required to setup an interaction with a black box to record the behaviour of the robot, but rarely this interaction is adequately supported, creating a burden for the teams. As described before, we developed a set of APIs that team members could use to interact with the Data Hub in a transparent way. On average, the team members appreciated the addition (3.69± 0.82) as a way to simplify the interaction. It is worth noting that the lowest scores were awarded by teams involved in “Episode 4—Take the elevator”, where the interaction with the Data Hub was minimal. This may have reduced the perceived utility of the APIs.

#### 5.2.7 Impact of the Data Hub on the Organisation of the Competition

In SciRoc 2019, most of the referees were also directly involved in the organisation of the competition and the development of the rulebooks. Therefore, we asked them to rate how difficult it was to integrate the interaction with the Data Hub in the competition, from the point of view of the organisation. On average, the organisers felt that the impact was neutral (3.00± 0.93), making the design of the competition neither easier nor harder with the addition of the Data Hub. This is a success, because it means the integration was seamless with respect to the existing definitions.

#### 5.2.8 Impact of the Data Hub on the Competition Rules

The definition of the rulebooks is an element of the organisation of the competition that impacts on both the referees and the teams. Indeed, it is challenging for the organisers to define clear and understandable rules and for the teams to succeed in getting all the necessary information from the rulebooks. We asked two questions about clarity and difficulty of the rules, and how they were impacted by the addition of the Data Hub. For both questions the result were very similar (3.22± 0.74 for clarity, and 3.44± 0.83 for difficulty) and showed that the Data Hub had a neutral impact on the rules. Moreover, both referees and teams gave a very similar feedback. As for the general definition, also in this case a neutral impact is a success, since it means that the Data Hub was seamlessly integrated with the rules.

#### 5.2.9 Interfacing the Data Hub With the Robots

Robot competitions are extremely challenging development-wise for the teams involved, since some of the problems have to be solved directly during the competition days. For this reason, we implemented the APIs to interface the robot to the Data Hub. We asked the teams to express how difficult they felt was the integration. On average, we received a positive feedback (3.44± 0.82), with no team rating the integration as “very hard”.

The relatively high variability of the answers can be traced back to the personal skills of each team member. Robotics is very multi-disciplinary and not all roboticists have a programming background. Additionally, while data exchanges are not included in critical execution loops (e.g., navigation, obstacle avoidance), robot actions were often triggered by interactions with the Data Hub. Therefore, difficulties may arise, depending on the way the communication is implemented within the software of the robots. This dependency may be perceived as an added complexity by the developers.

#### 5.2.10 Novelty Introduced by the Data Hub

We asked both teams and referees to report if they felt the introduction of the Data Hub created an element of novelty in the definition of the challenges. The result obtained was quite interesting. Overall, 81.48% answered “Yes”, but if we take into account their roles in the competition, 100% of the teams perceived novelty, while only 64.29% of the referees answered positively. This is in line with the perceived neutral impact the Data Hub had in the definition of the rules. Additionally, some organisers deliberately tried to minimise the changes to their episode with respect to previous competitions, to streamline the definition process. All these elements together reduced the perceived novelty by the referees. Nevertheless, the integration of the smart city element and the interface with the Data Hub were enough to make every challenge novel to all the team members.

#### 5.2.11 Usefulness of the Data Hub Interaction

The interaction with the Data Hub is concertised in four tools: the map, the status message visualiser, the trial management interface, and the robot activity log. According to team members and referees, the most useful of these tools is the status message visualiser (4.32± 0.68). The result does not change when considering the two categories of participants separately. However, on average the team members gave slightly lower scores, albeit still positive (greater or equal to 4.00).

#### 5.2.12 Extension of the Data Hub Interaction

Almost unanimously all participants think it is worth to expand the Data Hub interaction for future competitions. We asked them to specify which element among explanation, monitoring, and reproducibility they would like to see improved. Explanation was the most requested additional feature with 76.92% of the participants selecting it, followed by monitoring, mentioned by 50% of them. In third place, reproducibility was selected in 42.31% of cases.

## 6 Research Directions

We now discuss the findings of our survey in the light of future directions for research. Explainable Artificial Intelligence (Adadi and Berrada, 2018; Došilović et al., 2018) is currently a very important topic in AI especially in the context of machine learning. Therefore, it was, unsurprisingly, considered the most important aspect in the questionnaire and the most requested feature to be extended. Robots are complex systems orchestrating multiple components (Bardaro et al., 2018), each one with a different level of explainability. For example, a robot can exploit deep learning to perform scene analysis, while using A* for navigation. This heterogeneous configuration combined with the complexity introduced by the interaction of the different components make system-level robotic explainability a difficult task. The main challenge is to manage the many data streams exchanged between components (e.g., sensor data, control commands, navigation plans, manipulation tasks, etc.) and understand how they impact the execution of a specific functionality.

Currently, robotics tend to gravitate towards two opposite approaches when tackling this challenge. In one case, the robot exposes every input and output it processes. Then, a robotic expert uses their knowledge to understand which is relevant to a specific situation. For instance, this is the approach used by the tools provided by ROS. Here the explainability of the whole system is delegated to single components, and additional effort is needed to capture and explain the general behaviour of the robot. Alternatively, a selection of high-level reports is tailored around specific functionalities to provide a focused overview of the robot behaviour. Usually, this is the solution used to explain the robot behaviour to non-experts. However, it often lacks depth since it focuses on a few elements of the whole system and requires specific development effort for each of the presented functionalities.

Thanks to the features of the Data Hub and the contained environment created by a competition, we now have the possibility to introduce a solution that reaches a middle ground between these two options. On the technological side, the Data Hub provides an efficient and low-overhead (for the developer) solution to collect, store, and review the data produced by the robot. Differently from other solutions, the Data Hub is not specifically designed to interface with robots, but it is flexible and adaptable to multiple data producers. This means that it can be used to contextualise the robot behaviour with additional data sources. This was done in SciRoc 2019 on a small scale, where the updates coming from the robots (i.e., locations and status messages) were paired with episode specific data (e.g., inventory status, table configuration), and competition related information (e.g., trial results). On the adoption side, a competition gives the organisers the leverage to require the use of a specific set of tools and to expose all the necessary information for evaluating the performance of the robots. Moreover, the interaction with the Data Hub gives additional motivation to the teams to achieve solutions that are more robust and general. While this seems counter intuitive, because the developers should spontaneously adopt practices that are beneficial, it is supported by the results of the questionnaire.

Explanation is tied to the benchmarking capabilities that are at the core of robotics competitions. For this reason, it is an aspect that is highly requested not only by developers, but also by organisers. Many elements are necessary to correctly benchmark the performance of a robot. A competition provides a repeatable scenario and a set of requirements and achievements that can be used as a milestone-based performance metric to evaluate the behaviour of a robot. However, we could exploit the functionalities of the Data Hub to move beyond this type of evaluation, and increase the granularity of the metrics. For instance, with the current setup, in a scenario such as the one presented in “Episode 10—Trough the door”, it is impossible to differentiate between a robot that has the complete functionality of operating the door and one that is programmed to solve only that specific configuration. Indeed, with a more detailed introspection of the activities of the robot, it would be possible to identify the level of autonomy and adaptability of the platform without resorting to multiple runs of a dynamic benchmark.

The integration of a Data Hub in the SciRoc competition allowed us to highlight the need for a more principled support in the development of integrated service robots. In the future, we want to focus our attention on developing a Data Hub-driven explanation component for ROS to support explainable applications of service robots.

Another possible research direction revolves around using the competition as a testbed for security in a smart city. In the context of SciRoc, there was no need to prevent malicious intents from the participants, and we only implemented the necessary security to avoid accidental disruption between the teams. This design also reduced the teams’ development burden and maintained the focus on robotic applications. Nonetheless, if robots aim at becoming digital citizens of the smart city, it is crucial to guarantee they cannot become compromised (e.g., by granting access to their sensors to unauthorised users).

In recent years, the rising number of devices interacting with the smart city has become incompatible with a centralised architecture used to identify, authenticate, and connect all of them. Therefore, distributed technologies have become increasingly popular in this context (Xie et al., 2019). An example is the use of blockchain to manage access and authentication in an IoT infrastructure (Ouaddah et al., 2016). This approach can guarantee control and ownership of the data streams produced by the devices. Data ownership is important also in the contained scenario of a robotic competition since it can be used to register robot logs to support trustable auditing during post-challenge analysis. Additionally, the blockchain can enhance security in the communication among devices in the IoT network (Samaniego and Deters, 2017). While inter-agent communication is not part of the competition, it is a common real-world scenario and could provide a base to design multi-agents challenges in the future.

## 7 Conclusion

In the 2019 edition of the Smart Cities and Robotic Challenge we played a double role: we were local organisers as well as in charge of the integration of the smart city elements within the competition. We fulfilled this brief by capitalising on the MK Data Hub infrastructure, which acted as an interface to a simulated smart city.

We extended the design of the original challenges defined by the European Robotics League to include smart city elements, such as notifying a centralised system of the status and position of the robots, relying on external information and resources to complete tasks, and maintaining consistency between robots observations and remote datasets. We also developed a set of tools supported by the Data Hub, to facilitate the interaction between the robots and the smart city elements (i.e., remote APIs) and to support the activities of the referees during the competition (i.e., trial management interface). Thanks to this setup, it was also possible to realise an engaging experience for the general public and increase their understanding of robotic tasks.

Overall, our contribution to SciRoc 2019 has been received positively by all the actors involved in the experience. The results of the survey show that the general public felt engagement with the tasks executed by the robots. The members of the competing teams also reacted positively to the experience: while they were reluctant at first to integrate our technologies in their applications, as shown by the neutral feedback in the questionnaire, they ultimately appreciated the tools we provided and the additional challenges enabled by the smart city element. Lastly, referees and organisers were very supportive of the use of screens and tailored messages, both for general public engagement and to support robot evaluation.

The work done in SciRoc 2019 has allowed us to explore the integration of robots and smart cities in a controlled environment. We proposed a series of tools to facilitate this integration and we were able to gauge which aspects of our work were more impactful. Nevertheless, there is still a long way to go to make robot competitions and robotics in general accessible and understandable by the general public. In the future, we will continue to work alongside robotic competitions and in particular we plan to extend our tools to strengthen the explanation aspect. To this purpose, we will work on a more seamless integration with existing robotic software frameworks, such as ROS, to facilitate the management and recording of the many data streams generated by the robot. Additionally, we will extend the tools developed to support the competition to provide more functionalities to aggregate, visualise, and analyse the data produced by the robots and collected by the Data Hub.

## Data Availability

The original contributions presented in the study are included in the article/Supplementary Material, further inquiries can be directed to the corresponding author.

## Footnotes

1
http://www.mksmart.org/

2
https://datahub.mksmart.org/demos/sciroc-replay/

3
https://bit.ly/3oR3yXT

4 The full questionnaire is available here: https://www.cognitoforms.com/KMi6/sciroc2019. Additionally, a text version is in Supplementary Table S1.

5 Full results are available in Supplementary Table S2.
