# Supplementary material for: Introducing a Smart City Component in a Robotic Competition: A Field Report
Source: Front Robot AI. 2022 Feb 16;9:728628. doi: 10.3389/frobt.2022.728628 (PMC8888918; doi:10.3389/frobt.2022.728628)
Supplement: Supplementary file 1 [file Table1.pdf]

SciRoc 2019 Questionnaire

This table contains a text version of the questionnaire used for the survey. Each question specifies the type of answer used. When a Likert scale is used it is always between 1 and 5.

Questionnaire - Part 1

- What was your role during the SciRoc competition in Milton Keynes? (**multiple choice**)
  - I was a Referee, Assistant referee, or a member of the Organisation
  - I was a member of one of the teams taking part in the challenge
  - I was a Volunteer helping with performing the trials
- What episode(s) you were mainly involved in? (**checkbox**)
- How do you grade the level of engagement of the general public during the competition? (**Likert scale**)
- How do you grade the importance of each aspect (i.e., explanation, monitoring, reproducibility) in the context of robots in cities? (**Likert scale**)
- How do you grade the support of each aspect (i.e., explanation, monitoring, reproducibility) during the SciRoc competition? (**Likert scale**)
- The on-site screens have been helpful in engaging the visitors. (**Likert scale**)

Questionnaire - Part 2 - Referee

- The on-site screens have been useful in supporting Referees (**Likert scale**)
- The trials management interface has been useful in supporting Referees (**Likert scale**)
- How difficult it was to organise the competition considering the addition of the DataHub? (**Likert scale**)
- The Data Hub helped to improve the quality of competition rules (**Likert scale**)
- The Data Hub made more difficult the definition/understanding of competition rules (**Likert scale**)
- Did the Data Hub open new opportunities in designing novel type of tasks? (Yes/No)
- Which component of the Robot-Data Hub interaction did you find more useful?
  - Maps on screens (**Likert scale**)
  - Status messages (**Likert scale**)
  - Trial management interface (**Likert scale**)
  - Robot activity log + Video (**Likert scale**)
- Do you think is it worth to expand the approach in future competitions? (**Yes/No**)
- In which direction (**checkbox**)
  - More explanation
  - More monitoring
  - More reproducibility
  - Other

Questionnaire - Part 2 - Competitors

- The on-site screens have been useful in supporting Competitors (**Likert scale**)
- The data management APIs have been useful in supporting Competitors (**Likert scale**)
- How difficult it was integrating the robot abilities with the DataHub? (**Likert scale**)
- The Data Hub helped to improve the quality of competition rules (**Likert scale**)
- The Data Hub made more difficult the definition/understanding of competition rules (**Likert scale**)
- Did the Data Hub open new opportunities in designing novel type of tasks? (**Yes/No**)
- Which component of the Robot-Data Hub interaction did you find more useful?
  - Maps on screens (**Likert scale**)
  - Status messages (**Likert scale**)
  - Trial management interface (**Likert scale**)
  - Robot activity log + Video (**Likert scale**)
- Do you think is it worth to expand the approach in future competitions? (Yes/No)
- In which direction (**checkbox**)
  - More explanation
  - More monitoring
  - More reproducibility
  - Other
