# Supplementary material for: Introducing a Smart City Component in a Robotic Competition: A Field Report
Source: Front Robot AI. 2022 Feb 16;9:728628. doi: 10.3389/frobt.2022.728628 (PMC8888918; doi:10.3389/frobt.2022.728628)
Supplement: Supplementary file 2 [file Table2.pdf]

What Was Your Role	Episodes	Engagement General Public	Importance Of Explanation	Importance Of Monitoring	Importance Of Reproducibility	Support Of Explanation	Support Of Monitoring	Support Of Reproducibility	Usefulness Of Screens	Usefulness Of TMI	Usefulness Of APIs	Difficulty With Addition Of DH	DH Improve The Rules	Difficulty Integration DH	DH More Difficult Rules	Novelty with DH	More Useful: Maps	More Useful: Status Messages	More Useful: TMI	More Useful: Robot Activity Log Video	Expand The Approach	Explanation	Monitoring	Reproducibility	InWhichDirection
Referee	3	3,00	4,00	4,00	4,00	4,00	4,00	3,00	3,00	3,00		3,00	2,00		2,00	No	4,00	4,00	3,00	3,00	No	☐	☐	☐	
Referee	3	5,00	5,00	5,00	5,00	4,00	5,00	3,00	4,00	3,00		3,00	3,00		3,00	Yes	5,00	4,00	5,00		Yes	☑	☑	☐	More explanation, More monitoring
Referee	3	5,00	5,00	5,00	3,00	4,00	4,00	4,00	4,00	4,00		3,00	2,00		2,00	Yes	4,00	5,00	4,00	4,00	Yes	☑	☐	☐	More explanation
Referee	3	4,00	5,00	4,00	4,00	4,00	4,00	4,00	5,00	4,00		4,00	2,00		4,00	Yes	5,00	5,00	3,00	3,00	Yes	☐	☑	☐	More monitoring
Referee	4	4,00	3,00	4,00	5,00	4,00	4,00	4,00	5,00	4,00		5,00	3,00		2,00	Yes	3,00	4,00	4,00	4,00	Yes	☐	☐	☑	More reproducibility
Referee	4	4,00	5,00	5,00	4,00	4,00	4,00	3,00	5,00	4,00		3,00	3,00		3,00	Yes	5,00	4,00	4,00	5,00	Yes	☑	☑	☑	More explanation, More monitoring, More reproducibility
Referee	7	3,00	5,00	4,00	5,00	4,00	4,00	2,00	3,00	4,00		2,00	4,00		2,00	Yes	2,00	5,00	4,00		Yes	☑	☐	☑	More reproducibility, More explanation, Other
Referee	7	3,00	4,00	4,00	4,00	3,00	3,00	3,00	4,00	4,00		3,00	3,00		2,00	No	3,00	4,00	4,00	5,00	Yes	☑	☑	☐	More explanation, More monitoring
Referee	10	5,00	5,00	5,00	5,00	5,00	5,00	5,00	5,00	5,00		4,00	4,00		2,00	Yes		5,00	5,00	5,00	Yes	☑	☑	☑	More explanation, More monitoring, More reproducibility
Referee	10	5,00	4,00	3,00	2,00	2,00	5,00	3,00	3,00	3,00		3,00	4,00		2,00	No	4,00	3,00	3,00	5,00	Yes	☑	☐	☐	More explanation
Referee	10	4,00	5,00	4,00	4,00	3,00	4,00	2,00	4,00	4,00		3,00	2,00		2,00	No	4,00	5,00	4,00	3,00	Yes	☑	☐	☐	More explanation
Referee	12	4,00	5,00	5,00	5,00	4,00	5,00	3,00	5,00	5,00		1,00	4,00		1,00	Yes	5,00	5,00	4,00	5,00	Yes	☑	☑	☐	More explanation, More monitoring
Referee	12	4,00	5,00	5,00	5,00	4,00	4,00	2,00	5,00	4,00		3,00	4,00		3,00	Yes	5,00	5,00	5,00	5,00	Yes	☑	☐	☑	More reproducibility, More explanation
Referee	12	3,00	4,00	3,00	5,00	5,00	5,00	4,00	3,00	3,00		2,00	4,00		2,00	No	5,00	5,00	4,00	4,00	Yes	☐	☐	☑	More reproducibility
Team	12	4,00	5,00	5,00	5,00	4,00	4,00	3,00	4,00		3,00		3,00	4,00	3,00	Yes	4,00	4,00	3,00	4,00	Yes	☐	☐	☐	Other
Team	3	5,00	4,00	5,00	4,00	4,00	5,00	4,00	4,00		4,00		3,00	2,00	2,00	Yes	5,00	4,00	5,00	5,00	Yes	☐	☑	☐	More monitoring
Team	3	5,00	4,00	3,00	5,00	3,00	3,00	5,00	2,00		3,00		4,00	1,00	1,00	Yes	3,00	3,00	4,00	4,00	Yes	☑	☐	☐	More explanation
Team	3	4,00	5,00	5,00	4,00	4,00	4,00	4,00	5,00		5,00		4,00	2,00	2,00	Yes	4,00	5,00	4,00	4,00	Yes	☑	☐	☐	More explanation
Team	3, 4	3,00	5,00	5,00	5,00	4,00	4,00	4,00	2,00		2,00		2,00	3,00	3,00	Yes				5,00	Yes	☑	☐	☑	More explanation, More reproducibility
Team	3, 4	4,00	5,00	5,00	5,00	4,00	4,00	4,00	4,00		4,00		4,00	2,00	2,00	Yes	5,00	5,00	4,00	5,00	Yes	☐	☐	☑	More reproducibility
Team	4	5,00	5,00	5,00	5,00	5,00	5,00	5,00	5,00		5,00		3,00	4,00	4,00	Yes	4,00	5,00	4,00	5,00	Yes	☑	☑	☑	More explanation, More monitoring, More reproducibility
Team	3, 4	2,00	4,00	4,00	4,00	2,00	2,00	4,00	3,00		3,00		3,00	3,00	4,00	Yes	3,00	4,00	3,00	4,00	Yes	☑	☑	☐	More monitoring, More explanation
Team	3, 4	5,00	4,00	4,00	3,00	4,00	5,00	4,00	5,00		4,00		3,00	3,00	3,00	Yes	5,00	4,00	3,00	3,00	Yes	☑	☑	☐	More explanation, More monitoring
Team	7	4,00	4,00	4,00	4,00	4,00	5,00	4,00	4,00		4,00		4,00	3,00	3,00	Yes	4,00	4,00	3,00	3,00	Yes	☑	☑	☐	More monitoring, More explanation
Team	3, 4	4,00	4,00	4,00	4,00	4,00	4,00	4,00	4,00		4,00		4,00	3,00	4,00	Yes	3,00	3,00	3,00	3,00	Yes	☑	☑	☑	More explanation, More monitoring, More reproducibility
Team	3, 4	4,00	4,00	4,00	4,00	4,00	4,00	4,00	4,00		4,00		3,00	3,00	3,00	Yes					Yes	☑	☑	☑	More explanation, More monitoring, More reproducibility
Team	3, 4	4,00	4,00	4,00	3,00	3,00	4,00	3,00	3,00		3,00		3,00	2,00	3,00	Yes	4,00	4,00	3,00	3,00	Yes	☑	☐	☐	More explanation
Volunteer	3, 4	4,00	5,00	4,00	5,00	2,00	2,00	2,00	3,00																
Volunteer	3, 4	4,00	5,00	3,00	5,00	4,00	3,00	4,00	2,00																
Volunteer	3, 4	4,00	5,00	4,00	4,00	4,00	4,00	4,00	4,00																
Volunteer	3	4,00	4,00	4,00	4,00	4,00	4,00	4,00	4,00																
Volunteer	4	5,00	3,00	3,00	3,00	3,00	3,00	3,00	5,00																
Volunteer	3	4,00	3,00	5,00	5,00	4,00	4,00	4,00	3,00																
Volunteer	4	2,00	3,00	3,00	3,00	2,00	2,00	2,00	3,00																
Volunteer	3, 4	4,00	4,00	5,00	4,00	4,00	3,00	4,00	4,00																
Volunteer	3, 4	4,00	4,00	3,00	3,00	4,00	4,00	4,00	3,00																

											Reversed		Reversed	Reversed									
AVG Global	4,00	4,36	4,19	4,17	3,72	3,94	3,56	3,83	3,86	3,69	3,00	3,22	3,31	3,44	81,48%	4,08	4,32	3,80	4,13	96,30%	76,92%	50,00%	42,31%
STDEV Global	0,78	0,67	0,74	0,80	0,77	0,85	0,83	0,93	0,64	0,82	0,93	0,74	0,82	0,83		0,86	0,68	0,69	0,83				
AVG Referee	4,00	4,57	4,29	4,21	3,86	4,29	3,21	3,82	3,86		3,00	3,14		3,71	64,29%	4,15	4,50	4,00	4,25	92,86%			
STDEV Referee	0,76	0,62	0,70	0,86	0,74	0,59	0,86	0,83	0,64		0,93	0,83		0,70		0,95	0,63	0,65	0,83				
AVG Team	4,08	4,38	4,38	4,23	3,77	4,08	4,00	3,77		3,69		3,31	3,31	3,15	100,00%	4,00	4,09	3,55	4,00	100,00%			
STDEV Team	0,83	0,49	0,62	0,70	0,70	0,83	0,55	0,97		0,82		0,61	0,82	0,86		0,74	0,67	0,66	0,82				
AVG Volunteer	3,89	4,00	3,78	4,00	3,44	3,22	3,44	3,44															
STDEV Volunteer	0,74	0,82	0,79	0,82	0,83	0,79	0,83	0,83															
